# CLRD: Collaborative Learning for Retinopathy Detection Using Fundus Images

**DOI:** 10.3390/bioengineering10080978

**Published:** 2023-08-18

**Authors:** Yuan Gao, Chenbin Ma, Lishuang Guo, Xuxiang Zhang, Xunming Ji

**Affiliations:** 1 Department of Biomedical Engineering, School of Biological Science and Medical Engineering, Beihang University, Beijing 100191, China; 2 Shen Yuan Honors College, Beihang University, Beijing 100191, China; 3 Department of Ophthalmology, Beijing Tiantan Hospital, Capital Medical University, Beijing 100050, China

**Keywords:** deep learning, collaborative learning, retinopathy detection, online distillation, fundus image

## Abstract

Retinopathy, a prevalent disease causing visual impairment and sometimes blindness, affects many individuals in the population. Early detection and treatment of the disease can be facilitated by monitoring the retina using fundus imaging. Nonetheless, the limited availability of fundus images and the imbalanced datasets warrant the development of more precise and efficient algorithms to enhance diagnostic performance. This study presents a novel online knowledge distillation framework, called CLRD, which employs a collaborative learning approach for detecting retinopathy. By combining student models with varying scales and architectures, the CLRD framework extracts crucial pathological information from fundus images. The transfer of knowledge is accomplished by developing distortion information particular to fundus images, thereby enhancing model invariance. Our selection of student models includes the Transformer-based BEiT and the CNN-based ConvNeXt, which achieve accuracies of 98.77% and 96.88%, respectively. Furthermore, the proposed method has 5.69–23.13%, 5.37–23.73%, 5.74–23.17%, 11.24–45.21%, and 5.87–24.96% higher accuracy, precision, recall, specificity, and F1 score, respectively, compared to the advanced visual model. The results of our study indicate that the CLRD framework can effectively minimize generalization errors without compromising independent predictions made by student models, offering novel directions for further investigations into detecting retinopathy.

## 1. Introduction

Every year, over 36 million people die due to chronic diseases, which account for more than 60% of all deaths [1]. These chronic diseases, including coronary artery disease, hypertension, and diabetes mellitus, frequently lead to vascular retinopathy. It is estimated that 233 million diabetic patients will suffer from retinopathy by the year 2040 [2]. At present, the detection method necessitates the manual inspection of fundus images to identify the presence of the disease. However, this detection approach is considerably time-consuming and requires a significant number of healthcare professionals, resulting in delayed medical treatment for many patients. Despite doctors recommending regular retinal check-ups for diabetic patients, several cases remain undetected until the disease has advanced [3]. Therefore, an automated system is essential for detecting retinopathy.

The continuous development of artificial intelligence technologies, such as machine learning [4,5,6,7,8] and deep learning [9,10,11], has made the high-performance detection of retinopathy possible. Traditional machine learning methods have been widely applied in this field. For example, Latha et al. [4] introduced an efficient Splat feature classification method for detecting retinopathy features, including hemorrhages. This method improved data usability by performing operations like denoising, morphological processing, and dynamic thresholding on fundus image data. Subsequently, supervised learning methods were employed to implement retinopathy detection, and feature extraction was directly used to process retinal hemorrhages. The results demonstrated the improved recall (REC) and specificity (SPE) of the model, with an area under the curve (AUC) of 96.00%, surpassing the previous model’s implementation of 87.00%. Furthermore, Marin et al. [5] screened for retinopathy, like diabetic macular edema and hard exudates, and obtained a set of candidate regions. They compared these regions with the results of support vector machine (SVM), multilayer perceptron, and K-nearest neighbor algorithms to validate them. Superior REC and SPE were achieved on a private dataset, and the classification results were very similar to those of ophthalmologists. Anton et al. [6] combined SVM and differential evolution algorithms for retinopathy detection and extracted highly relevant characteristics of retinal features, such as microaneurysms, hemorrhages, and neovascularization using an optimization algorithm. The algorithm achieved superior diagnostic accuracy (ACC) on the dataset, reaching 95.23%, and demonstrating its effectiveness in retinopathy detection. In addition, Haloi et al. [7] proposed a new algorithm for detecting microaneurysms in fundus images utilizing a local feature extraction algorithm. After preprocessing the fundus image data, the algorithm classified each pixel in the image as a microaneurysm or non-microaneurysm. The extracted features were then trained and tested using a model. The experiment demonstrated the superior classification performance of this method on a public dataset, with a REC of 96.54%. Finally, Kandhasamy et al. [8] used an SVM with selectively extracted features combined with genetic algorithms to perform clustering using mathematical morphology operations. The results were then passed to a multilevel set segmentation algorithm to statistically analyze the texture features of fundus images. The extracted features were finally classified using an SVM. Experimental validation showed excellent classification results for this algorithm.

Deep learning has emerged as the primary approach for detecting retinopathy, complementing conventional machine learning techniques. For example, Krishnan et al. [9] proposed a method that used convolutional neural networks (CNNs) and transfer learning to detect such lesions. They evaluated the performance of classic network models such as ResNet and InceptionResNetV2 by measuring the quadratic weighted Kappa values of different CNN architectures. Subsequently, they selectively fused the features of the best-performing models. The algorithm was validated on publicly available datasets, achieving a quadratic weighted Kappa value of 0.76, and thereby confirming the effectiveness of their proposed retinal detection algorithm. Similarly, Andronic et al. [10] constructed a CNN for diagnosing retinopathy and compared it with various classic neural network architectures such as ConvNets, GoogleNet, InceptionV4, and ResNeXT. Their proposed algorithm was also validated on public datasets, achieving a quadratic weighted Kappa value of 0.786, thus demonstrating superior classification performance. Moreover, Jiang et al. [11] preprocessed fundus images, then used transfer learning and a composite scaling model to detect retinopathy. They resolved the vanishing gradient problem caused by excessively deep models by introducing residual modules, allowing the model to focus more on regions with richer information features. Experimental results on public datasets verified that their proposed algorithm outperformed many advanced models currently in use.

Despite achieving superior classification performance, many retinopathy detection algorithms still fall short of meeting clinical practice requirements regarding diagnostic ACC and precision (PRE). Traditional image feature extraction algorithms can extract targeted lesion features but require professional medical knowledge [4,5,6,7,8]. Conversely, traditional direct image feature extraction algorithms have a stronger generalization ability but cannot accurately detect the specific scope of lesions, resulting in insufficient diagnostic ACC [4,5,6,7,8]. Artificial intelligence diagnostic algorithms based on neural networks require a large number of labeled sample data and massive computing resources [9,10,11]. A scarcity of samples and inadequate device computing power greatly affect the classification performance of a model. Moreover, overly complex network models and large input images increase the parameter quantity and computational cost, prolonging the model training time, thereby negatively impacting the diagnostic efficiency of retinopathy detection models [9,10,11].

Thus, we first introduce CLRD, a collaborative learning-based online knowledge distillation framework. The objective of this framework is to enhance the utilization of fundus images and improve the accuracy of the model diagnosis while reducing the model’s running time, thereby optimizing the overall diagnostic performance. Collaborative learning strategies are employed to integrate student models of various scales and architectures, enabling the extraction of valuable pathological information from fundus images. Knowledge is transferred through the design of distortion information that is tailored to fundus images, enhancing the model’s invariance. In this study, Transformer-based BEiT [12] and CNN-based ConvNeXt [13] are selected as student models. The research results demonstrate that the CLRD framework can significantly reduce generalization errors while maintaining independent predictions of student models, offering new avenues for future research in retinopathy detection.

## 2. Methodology

### 2.1. Problem Formulation

**Figure 1:**
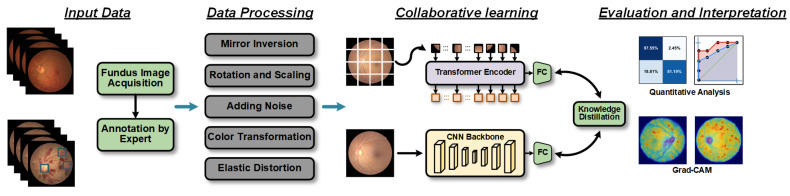
Workflow diagram of the proposed retinopathy detection method. First, fundus images are acquired, and the results labeled by experts are used as input data. Then, the fundus images are processed using five data augmentation techniques. Subsequently, the processed data are input into an online knowledge distillation framework based on collaborative learning strategies. Finally, the effectiveness of the method is verified and evaluated through quantitative evaluation and interpretability analysis.

The retinopathy detection problem refers to the analysis of fundus images to detect abnormal changes in the retina, and the overall workflow is shown in Figure 1. The paired sample consists of the input fundus image $X={\left\{x_{i}\right\}}_{i=1}^{n}\in {\mathbb{R}}^{w\times h\times c}$ and the corresponding target label $Y\in {\mathbb{R}}^{n\times 2}$ (i.e., the presence of retinopathy). To be precise, the width, height, and number of channels of any input fundus image *x_i_* are *w*, *h*, and *c*. Our proposed CLRD model learns the mapping from fundus images to the corresponding labels as follows:

(1) $$F:G_{l}({\left\{x_{i}\right\}}_{i=1}^{n})\to {\left\{y_{i}\right\}}_{i=1}^{n}$$

where F(·) denotes the objective optimization function for multiple $G_{l}(\cdot )$ to perform collaborative learning, and $G_{l}(\cdot )$ denotes the optimization function for the *l*-th feature extractor (student model). Mathematically, it can be described by minimizing the estimation error between the reference and output retinopathy labels.

### 2.2. Collaborative Learning for Knowledge Distillation

#### 2.2.1. Motivation

Knowledge distillation is a technique for reducing the complexity of neural networks [14]. It involves training a smaller student network with guidance from a larger teacher network, which improves the ability of the student network to detect retinopathy. The method achieves this by using a softened loss function based on Kullback–Leibler (KL) divergence between the outputs of the two networks [14]:

(2) $$L_{KD}=\frac{1}{b}\sum_{i=1}^{b}T^{2}KL\left(S(\frac{z_{t}}{T}),S(\frac{z_{s}}{T})\right)$$

where *b* is the batch size; ***z****_t_* and ***z****_s_* are logit of the teacher and student, respectively; *T* is the temperature parameter. S(·) is the softmax function, representing the softened probability distribution produced by the teacher and student.

To successfully apply this approach, a high-quality teacher network is critical. If the teacher network is not properly optimized or does not provide clear supervision, there will be a significant difference between soft and real targets. To measure the impact of different teacher models on student performance, we conducted experiments on a fundus image dataset using ResNet-18 as the student model. The performance of retinopathy detection was compared using different teacher models. All teacher models and student models were trained for 100 epochs. As shown in Table 1, teacher performance improved as the teacher size increased or when the representation architecture changed. This resulted in better supervision for the students, thereby improving their prediction performance. Therefore, we believe that online distillation using networks of different scales and architectures in a collaborative learning approach may have several advantages:

1. Improved feature extraction: CNNs can effectively extract local image features while vision Transformers (ViTs) capture global contextual information. Combining the two can improve retinopathy detection ACC through collaborative learning.
2. Multiscale feature learning: CNNs can extract features at different scales by using convolutional kernels of different sizes, whereas ViTs can capture long-range dependencies through self-attention mechanisms. Combining these two networks enables multiscale feature learning, which helps detect retinopathy of various sizes and shapes.
3. Increased model robustness: CNNs are invariant to image rotation and translation, whereas ViTs can fuse and learn multiscale features by introducing a multilayer, multiheaded attention mechanism. Using both networks together can maintain model stability when processing fundus images from heterogeneous sources.

#### 2.2.2. Overview

**Figure 2:**
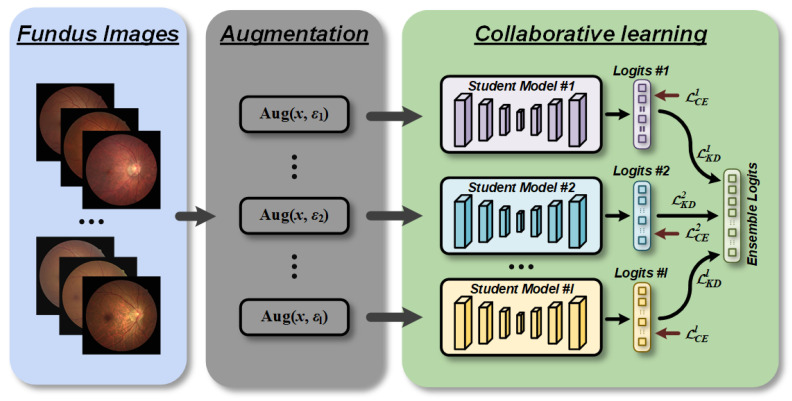
Overview of collaborative learning for retinopathy detection (CLRD) framework. We fed augmented fundus images controlled by different random seeds for each network separately to increase the invariance against disturbances in the data domain. The proposed CLRD dynamically integrates soft logits produced by student models of different architectures and scales through online distillation to continuously improve the performance of retinopathy detection.

Motivated by [14,17], we propose an approach for online automatic generation of soft targets to aid in the detection of retinopathy. Our CLRD framework, depicted in Figure 2, can be seen as a unified network comprising multiple student sub-networks. To ensure collaborative learning and bolster network robustness against input perturbations, we utilized various random seeds to enhance fundus images and generate soft targets that supervised all networks. We also introduced methods for minimizing soft targets to ensure that students with varying abilities benefitted from the learning process. Importantly, our models can be independently predicted, thereby avoiding any additional computational costs during testing. By adopting this technique, we achieved more accurate retinopathy detection and improve diagnostic efficiency.

#### 2.2.3. Objective Function

To enhance the generalization performance of our retinopathy detection algorithm, we incorporated KL divergence loss [14] to refine the soft-target knowledge into each student model. Specifically, we fine-tuned the weights pretrained on the ImageNet dataset for each student model. In addition, we trained all models end-to-end using a multitask loss function, including standard cross-entropy loss, to further optimize the model performance:

(3) $$L_{CLRD}=\sum_{i-1}^{l}L_{CE}^{i}+\alpha L_{KD}^{i}$$

where *α* is the trade-off weight. This approach not only improves the reliability and robustness of the model, but also accelerates the training process and reduces its complexity.

#### 2.2.4. Minimize Logits

In our CLRD framework, all models are students, and supervision is generated by integrating the outputs of the students. Assuming there are *l* students, the logit of the k-th student is defined as ***z****_k_*. The teacher logit ***z****_t_* is expressed as

(4) $$z_{t}=H(z_{1},z_{2},\ldots ,z_{l})$$

where H(·) is a function that produces a higher quality logit compared to the student’s logit. Assuming that training samples and test samples follow the same distribution, model predictions with smaller losses on the training set encourage students to converge faster. A simple combination method is to choose the logit with the smallest cross-entropy loss among all students, which can be defined as

(5) $$z_{t}=H(z_{1},z_{2},\ldots ,z_{l})=z_{k},k=\underset{i}{\arg\min}L_{CE}(z_{i},y)$$

where ***y*** denotes one-hot label. Although this simple combination is easy to implement, the teacher’s logit quality is not enough. Here, we propose to use the probability distribution generated by the softmax function to reflect the difference between the values in the logit: let ***z****^r^* be the element corresponding to the target label *r* in the logit, define ***z****^r^* = ***z*** − ***z****^r^*, and then the *r*-th element of ***z****^r^* is 0 for all subnetworks. The cross-entropy loss with one-hot labels will decrease as the other elements in the logit become smaller. Then, a neat way to generate the teacher logit is to choose the smallest element of each row of the matrix $Z^{r}=(z_{1}^{r};z_{2}^{r};\ldots ;z_{l}^{r})$. More precisely, the teacher logic can be expressed as

(6) $$z_{t,j}=\min\{Z_{j,i}^{r}|i=1,2,\ldots ,l\}$$

where ***z****_t_*,*_j_* is the *j*-th element of soft target ***z****_t_* and $Z_{j,i}^{r}$ is the element of the *j*-th row and *i*-th column in ***Z****^r^*.

#### 2.2.5. Invariant Collaborative Learning

Variations in eye structure and differences in imaging equipment, shooting conditions, and image preprocessing methods may result in various issues such as noise, blurring, brightness inconsistencies, lack of contrast, artifacts, etc., in fundus images. These problems can hinder the learning process of neural networks designed for retinopathy detection based on fundus images, leading to a decline in their performance.

**Figure 3:**
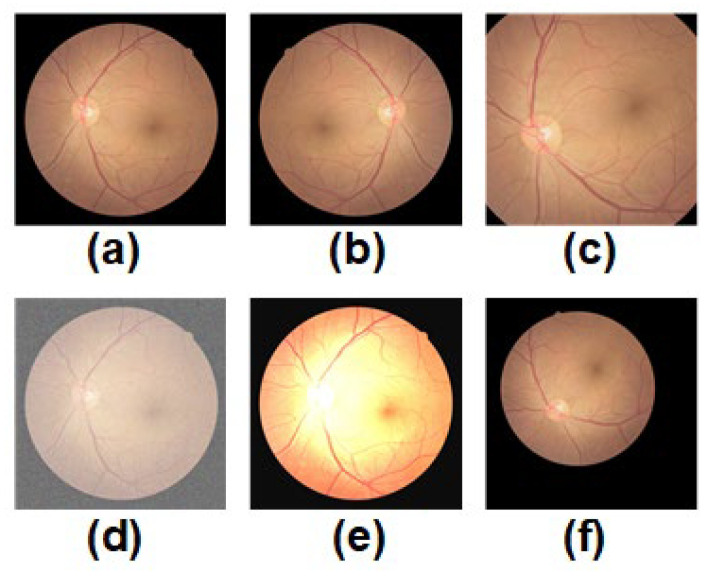
Schematic diagram of the five data augmentation techniques designed for fundus images used by CLRD is shown. Each of these techniques was applied independently to (**a**) a randomly selected original fundus image. These techniques include: (**b**) mirror inversion, (**c**) cropping and extraction of the region of interest, (**d**) adding noise, (**e**) color space transformation, and (**f**) elastic distortion.

To address these challenges effectively, we generated the same soft targets for all students containing similar distorted images. Furthermore, we employed the same data augmentation strategy for each student model by randomly selecting fundus images to integrate knowledge. It is important to note that we used only five techniques to ensure that the augmented fundus images retained the semantic information of the original fundus images, as shown in Figure 3. Here are the clinical implications of these data augmentation techniques:

1. Mirror inversion: This technique involves flipping an image from left to right, like seeing one’s reflection in a mirror. It can be used to study the symmetry of ocular diseases like retinal artery or vein angiomas.
2. Cropping and extraction of the region of interest: These techniques can change the size and angle of fundus images to better study the morphology and structure of various diseases. For instance, rotation and scaling techniques can help quantify the corneal curvature and angle when studying refractive errors.
3. Adding noise: Noise refers to random fluctuations or disturbances in an image that simulate various noisy situations in real life, such as poor lighting conditions or camera shake. This technique can improve the recognition performance of neural networks for under-eye images in noisy environments.
4. Color transformation: Color transformation techniques can adjust parameters like the color, brightness, and contrast of an image to better observe the details and structures of retinal diseases. For example, when studying retinal fissures, color transformation techniques can enhance specific areas around the fissure.
5. Elastic distortion: Elastic distortion techniques simulate eye deformation in different positions and orientations to study the morphology and structure of various ocular diseases better. For example, when studying the anterior macula, elastic distortion techniques can reconstruct the true shape and contour of the anterior macula.

Using these data augmentation techniques increases the amount and diversity of fundus images required for training, which can encourage student models with low generalization errors.

### 2.3. Feature Extractors

We conducted comparison experiments (as shown in Section 4.2) to identify the optimal two student models, which are briefly described below.

#### 2.3.1. BEiT

**Figure 4:**
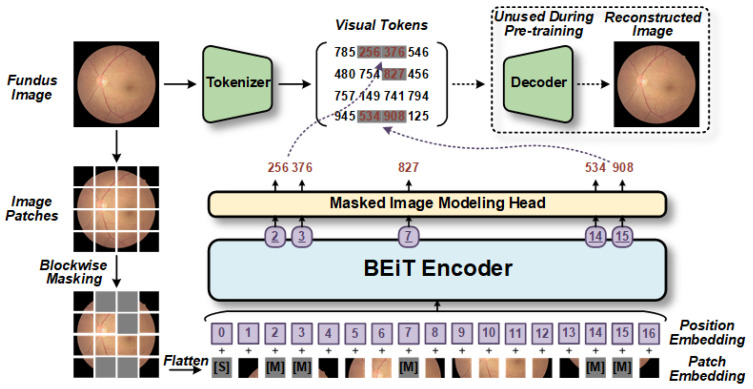
The BEiT model utilizes self-supervised pretraining [12]. Initially, the model randomly selects blocks of fundus images for block-wise masking and then performs position-embedding on the flattened blocks. These markers, which include position information, are fed into the Transformer-based encoder to generate a series of estimation vectors, with each corresponding to a masked block. Subsequently, these estimation vectors are inputted into the masked image modeling head based on the fully connected layer to predict the visual tokens generated by the tokenizer. Consequently, the optimized decoder learns better hidden variables to restore the original fundus image more accurately. [S] denotes the start token, while [M] denotes the masked token.

BEiT is a self-supervised visual representation model that can be utilized for retinopathy detection using fundus images [12]. Unlike traditional deep learning models, BEiT uses the Transformer as its backbone network. As shown in Figure 4, the pretraining task of BEiT is based on masked image modeling. In this approach, each image is partitioned into discrete tokens of visual information and transformed. Subsequently, self-supervised training is conducted on these tokens to reconstruct the original visual tokens on the corrupted image blocks. This process enhances the network’s comprehension of visual data. The pretraining approach of BEiT equips it with strong visual feature representation and generalization abilities. It can be fine-tuned in downstream tasks to adapt to different application scenarios. For instance, in retinopathy detection based on fundus images, the pretrained model of BEiT can be directly fine-tuned on the fundus images.

#### 2.3.2. ConvNeXt

We opted to use ConvNeXt [13] as the backbone model for detecting retinopathy due to its superior ACC and scalability over Vanilla ViT [16]. Despite being constructed entirely from CNNs, it maintains the simplicity and effectiveness of standard CNNs. The primary advantage of ConvNeXt is its ability to provide output features at different scales, which is critical for detecting small lesions in fundus images.

**Figure 5:**
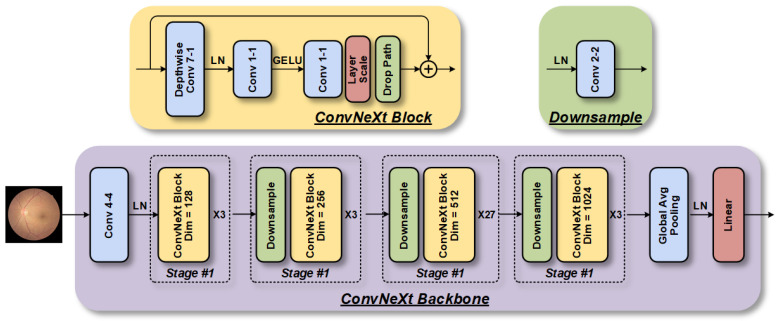
The ConvNeXt model, which is based on the CNN architecture, consists of four-stage feature extraction modules that are stacked to extract fundus image features at different scales.

The architecture of a ConvNeXt-based model for retinopathy detection comprises four stages, as shown in Figure 5, each containing a varying number of ConvNeXt blocks [13]. The initial downsampling module employs a Patchify layer that consists of a convolutional layer with a kernel size of 4 × 4. The ConvNeXt blocks possess a larger kernel size (7 × 7) and an inverted bottleneck structure that differs from ResNet. Furthermore, the commonly used batch normalization and ReLU activation functions in CNNs are substituted with layer normalization (LN) layers and Gaussian error linear unit (GELU) functions, employed by Transformers, respectively. Consequently, the number of layers is reduced, leading to a more efficient model. For stable training, a separate 2 × 2 convolutional downsampling layer with a step size of 2 is included, and an LN layer is added later. These alterations enhance the performance of the ConvNeXt model when applied to retinopathy detection.

## 3. Experimental Setup

### 3.1. Data Description

This study was conducted in adherence to the Helsinki Declaration and approved protocol (NO. 2022101) by the ethics committee, which included a waiver of informed consent as it poses minimal risk to patients’ health and rights. The Macula-centered fundus images were captured from the ophthalmology department of Xuanwu Hospital between 1 August 2017 and 1 March 2022. The raw dataset was comprised of 1521 images obtained from 1137 patients who visited the department for examinations. Typically, patients undergoing these examinations required retinal color fundus photographs that were obtained through pharmacological pupil dilation, with multiple images taken per eye. However, since the goal of this project was to screen preoperative fundus images and diagnose potential lesions, all postoperative fundus images were excluded. Detailed statistical information regarding the dataset can be found in Table 2.

### 3.2. Data Preprocessing

The intricacy of the retinal structure can often lead to confusion between retinopathy and other ocular diseases. Moreover, during our study, we encountered a multitude of imaging noises such as black spaces on both sides of the eye, low contrast, lens blurring, or insufficient lighting. As a result, the model failed to precisely identify minor fundus damage in the poorer quality photographs. Therefore, it was necessary to preprocess the images before conducting the study.

Initially, we designed an algorithm that effectively removed invalid black regions by cropping a fixed number of pixels from all four sides of each image, while avoiding any significant computational overhead caused by the black space. Subsequently, we normalized the resolutions of the original images, which varied from 2592 × 1728 to 3000 × 3000, to a uniform size, complying with the input requirements of the specific model. We also converted all images to grayscale to measure the light intensity of individual pixels in a single image. For images with an excessively bright or dark foreground and background, we employed histogram equalization to enhance visualization and discover hidden information. To improve the local contrast and enhance edge sharpness in the image regions, we used the adaptive histogram equalization method [18]. Additionally, for enhancing the contrast effect in each region of the image in dark images, we provided a contrast stretching algorithm, which is defined as follows:

(7) $$x_{i}(p,q)=\frac{x_{i}(p,q)-\min x_{i}}{\max x_{i}-\min x_{i}}\times 255$$

where *x_i_*(*p*, *q*) is the gray value of a certain pixel in the original fundus image, and min *x_i_* and max *x_i_* are the actual minimum and maximum gray values in the original fundus image, respectively.

### 3.3. Training and Validation

The CLRD was trained using a five-fold cross-validation paradigm, ensuring a robust assessment. The dataset was partitioned into five subsets, with four subsets (*n* = 1216) used for training and one subset (*n* = 305) for validation, allowing for a representative estimation of model performance while mitigating data variability. By maximizing the utilization of available data, reliable estimates of the model’s performance on unseen data were obtained. To maintain independence in the predictions and ensure a reliable performance assessment, we carefully assigned both eyes from the same patient to either the training set or the outcome set during dataset splitting. A similar five-fold cross-validation approach was employed to evaluate the generalization capability of our proposed method on the external test set, EyePACS. All models underwent 200 epochs of training with an initial learning rate at 1 × 10^−3^ and decreased by 0.1 at the 100th and 150th epochs. The weight decay was set to 5 × 10^−4^, batch size to 32, and momentum to 0.9. The temperature value for T was 2.

### 3.4. Evaluation Criteria

We used various quantitative metrics to assess the performance of the model in retinopathy detection. The ACC metric represented the proportion of samples that were correctly classified as either retinopathy or normal, while PRE was the proportion of true-positive samples among those detected as retinopathy. For early screening systems, REC and SPE served as crucial indicators to determine referral and directly indicated the effectiveness of retinopathy detection. REC measured the proportion of all retinopathy samples that were correctly predicted, while SPE represented the proportion of all normal samples that were correctly identified. To further evaluate the balance of the model between REC and SPE, we utilized the receiver operating characteristic (ROC) curve and its AUC to visualize model performance. The F1 score, which is the averaged sum of PRE and REC, indicated good retinopathy detection ACC when its value was close to 1. Additionally, to demonstrate the interpretability of the models, we employed the gradient-weighted class-activation-mapping (Grad-CAM) [19] method to visualize attention regions.

To evaluate model computational efficiency, we used the Params and FLOPs’ metrics. The Params metric represented the number of parameters in the model, which included the total number of weights and biases that need to be learned in the model. This metric is typically used to evaluate the size and storage requirements of the model. On the other hand, FLOPs referred to the floating-point operations per second, representing the computational complexity and speed of the model.

## 4. Results

### 4.1. Quantitative Analysis

**Figure 6:**
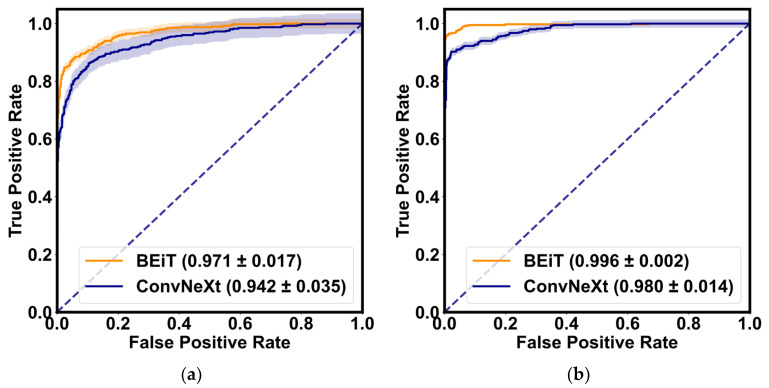
The ROC curves of the BEiT and ConvNeXt models for the retinopathy detection are shown, respectively. (**a**) ROC curve without collaborative learning; (**b**) ROC curve of the proposed CLRD. The solid line is the mean of the five-fold cross-validation, and the shaded part is the standard deviation.

**Figure 7:**
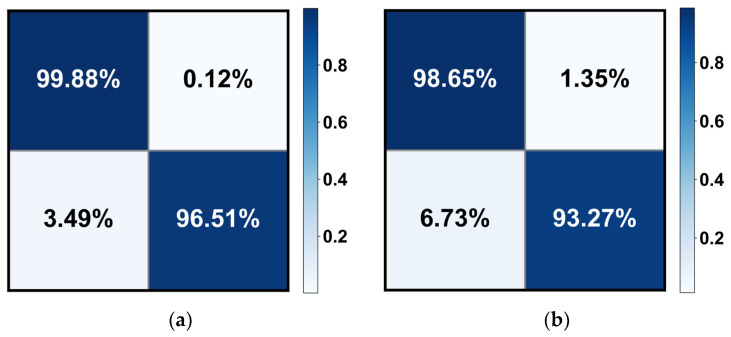
The confusion matrix of the (**a**) BEiT and (**b**) ConvNeXt models for retinopathy detection. Note that only the results of the median in the five-fold cross-validation are calculated here.

Table 3 demonstrates the quantitative evaluation results of the five-fold cross-validation of the CLRD student models, and Figure 6 and Figure 7 correspond to its ROC curve and confusion matrix. The experimental results show that the proposed CLRD has a high ACC in retinopathy detection. From the results, it can be seen that the average ACC, PRE, REC, SPE, and F1 scores of the student model based on the ConvNeXt architecture are 96.88%, 96.88%, 96.87%, 95.04%, and 96.86%, respectively. The model uses simple operations such as convolutional and pooling layers to extract features and is therefore more suitable for processing information with local relevance in fundus images. In contrast, BEiT introduces complex structures such as a self-attention mechanism and multiheaded attention mechanism, which can better handle data with global relationships. Therefore, the average ACC, PRE, REC, SPE, and F1 scores of the student model based on the BEiT architecture improved by 1.95%, 1.96%, 1.96%, 2.71%, and 1.96%, respectively. In addition, we found that the ROC curves of the BEiT model have lower standard deviations in the five-fold cross-validation, indicating that the self-supervised model based on the Transformer architecture has a more robust inference capability. Meanwhile, the confusion matrix confirms that the proposed CLRD framework can suppress the estimation bias caused by the imbalance of fundus images, thus improving the performance of the model.

### 4.2. Comparison with Different Architectures

We conducted a quantitative comparison of the feature extractor settings of different architectures in the student model of CLRD for retinopathy detection, with the aim of identifying the optimal architectural settings. In particular, we designed experiments using two types of CLRD for collaborative learning: CLRD-1, which replaces BEiT with Vanilla ViT; and CLRD-2, which replaces ConvNeXt with ResNetV2. Table 4 presents the experimental results, which show that although the large pretrained ResNetV2 architecture has the lowest Params and FLOPs, it is not as effective in collaborative learning as the ConvNeXt architecture.

In detail, we observed that in CLRD-1, the average ACC, PRE, REC, SPE, and F1 scores of BEiT decrease by 1.34%, 1.26%, 2.67%, 2.87%, and 1.36%, respectively. Similarly, in CLRD-2, Vanilla ViT’s Params and FLOPs are 1.69% lower than the recommended CLRD-3 regarding the F1 score, despite being 26.19% and 32.91% higher than BEiT, respectively. Moreover, we found that the average ACC, PRE, REC, SPE, and F1 scores of ConvNeXt for collaborative learning decrease by 1.28%, 1.05%, 3.46%, 3.84%, and 1.34%, respectively. Based on these findings, we can conclude that feature extractors should be chosen carefully in the CLRD framework, and that a retinopathy detection performance can be improved by pairing student models with different architectures and complexities.

### 4.3. Interpretability Analysis

**Figure 8:**
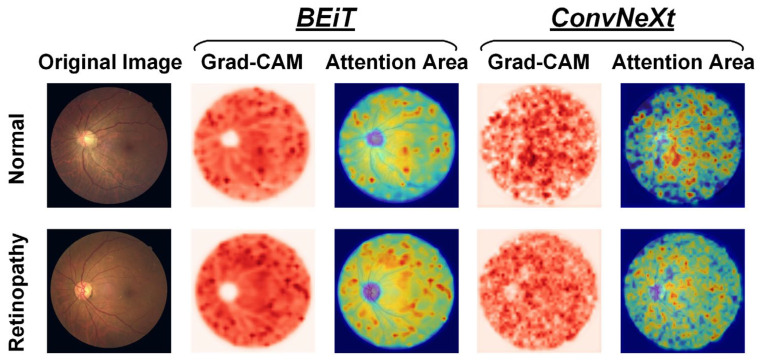
Visualization of attention regions in normal and retinopathy fundus images using two feature extractors in the proposed CLRD framework, respectively.

When visualizing the attention regions of a model through Grad-CAM technology, as depicted in Figure 8, it becomes evident that there are differences in the model performance. Broadly speaking, CNN-based ConvNeXt has a propensity to focus on discrete regions that cover retinal vessels and unrelated backgrounds, whereas Transformer-based BEiT predominately attends to continuous areas that reflect global features. In particular, the ConvNeXt model emphasizes the fine-grained characteristics of fundus images, such as microvascular distortions and hemorrhages within lesion areas, which aid in detecting local information, including changes in vessel morphology like thickness, curvature, and branching, among others, as well as abnormal structures that are often present in retinopathy, such as exudates, tissue proliferation, and atrophy. Conversely, the BEiT model places greater focus on the tissues outside the optic disc in retinopathy samples, thereby facilitating its ability to more efficiently capture the coarse-grained global characteristics of fundus images, including factors such as eye size and shape, vessel distribution, and pigment changes, among others. The incorporation of global information is particularly advantageous in improving the surveillance of widespread illnesses, such as through the prediction of epidemic infection trends of uveitis. Based on prior research related to retinopathy detection through fundus images, our proposed CLRD methodology effectively combines models of varying scales and structures to supply highly pertinent, detailed local and global features that are beneficial in detecting retinal diseases with increased ACC. In turn, this is essential for enabling earlier diagnoses and treatments of retinopathy.

### 4.4. Ablation Studies

#### 4.4.1. Sensitivity to Loss Hyperparameter

In all other experiments, we set the hyperparameter *α* to 1 to emphasize the effectiveness of DCLR. However, in this ablation experiment, we varied the *α* setting from 0.1 to 5 to conduct a sensitivity analysis of the loss hyperparameters. The experimental results are presented in Table 5. These results indicate that performance was not greatly impacted by *α* selections ranging from 0.2 to 1.5. Despite this, the careful adjustment of hyperparameters can lead to additional performance gains for retinopathy detection.

#### 4.4.2. Sensitivity to Data Augmentation Policies

To compare the performance differences of various data augmentation methods during model training and testing, we conducted sensitivity analysis experiments. These involved applying different data augmentation techniques to the same set of fundus images and comparing their results to determine the optimal method. The experimental results depicted in Table 6 indicate that our proposed data augmentation technique outperforms the others. Therefore, we posit that designing a specific data augmentation method for fundus images could lead to additional performance gains in retinopathy detection. However, when using only data augmentation methods derived from natural images (such as Cutout [20]), the ACC in retinopathy detection may be impaired due to a lack of support for clinical physiological significance.

## 5. Discussion

### 5.1. Comparison with Competitive Models

To demonstrate the effectiveness of the CLRD framework in facilitating deep neural networks for retinopathy detection, we replicated several state-of-the-art (SOTA) models. These models consisted of DenseNet121 [21], ResNetV2 [15], Xception71 [22], and EfficientNet [23], which are based on the CNN architecture, as well as Vanilla ViT [16], MobileViT [24], Swin Transformer [25], ConViT [26], CaiT [27], EfficientFormer [28], and VOLO [29], which are based on the Transformer architecture. We performed a quantitative comparison of these models using the same experimental setup, and the results are presented in Table 7. Our findings indicate that the proposed CLRD model achieved the highest ACC for retinopathy detection, surpassing all other models. Specifically, when compared to the second-best performing model, MobileViT, CLRD-BEiT improved the average ACC, PRE, REC, SPE, and F1 scores by 5.69%, 5.37%, 5.74%, 11.24%, and 5.87%, respectively. Notably, most competing models achieved F1 scores below 80.00%, thereby underscoring the significant role of the collaborative learning training paradigm in enhancing the ACC of retinopathy detection.

Compared to other models used for automated output, collaborative learning offers several key benefits. Firstly, it promotes model invariance by incorporating the knowledge transfer through distortion information specific to fundus images. This enables the CLRD framework to effectively minimize the generalization error, ensuring reliable and accurate predictions. In the context of retinopathy detection, this is crucial for making informed decisions and facilitating early treatment.

Secondly, collaborative learning facilitates independent predictions made by each student model while still benefiting from their collective knowledge. This allows the CLRD framework to take advantage of the unique insights offered by each model, leading to improved accuracy, precision, recall, specificity, and F1 score compared to advanced visual models. The superior performance of the CLRD framework demonstrates its potential as a way forward in enhancing the detection of retinopathy.

Furthermore, collaborative learning offers new directions for further investigations into detecting retinopathy. By exploring the combination of different models and architectures, we can continue to improve the diagnostic accuracy and efficiency of retinopathy detection algorithms.

### 5.2. Generalizability and Clinical Implications

We utilized the EyePACS fundus image dataset as an external test set to validate the generalization ability of our proposed CLRD method for retinopathy detection. In order to ensure a fair comparison, we conducted five-fold cross-validation experiments on this test set using the same parameter settings and presented the average performance of the five estimates in Table 8. Alongside this, we summarized the quantitative results from other SOTA work in Table 8. The results demonstrate that our CLRD method exhibits the most accurate performance in retinopathy detection. Compared to other methods, CLRD improved the ACC, PRE, REC, and F1 scores by 1.07% to 22.59%, 13.47% to 54.91%, 0.43% to 28.84%, and 0.23% to 83.02%, respectively. The CLRD method produces more reliable results as it employs collaborative learning of online distillation methods. This facilitates the efficient handling of unbalanced datasets, prevents overfitting, and ensures independent predictions.

Specifically, the presence of unbalanced EyePACS datasets often leads to models being biased towards predicting normal categories while ignoring abnormal retinopathy samples. Relying solely on ACC or reporting too few quantitative metrics is considered unreliable. For instance, the authors of [31] achieved an ACC of 87.37% and an F1 score of approximately 81.80% by calculating the entropy of each fundus image to highlight the lesion’s edge and creating regions of interest for the CNN model. However, this method disregards the imbalance of fundus images and employs only AUC as the core metric of performance. On the other hand, using an insufficient number of training samples carries the risk of overfitting. For instance, Xu et al. [30] employed only 360 fundus images as the training set for an eight-layer CNN model. Although they achieved a 94.50% ACC, the model’s generalization ability remained limited due to the small amount of data. To address these challenges, some researchers have utilized the model ensemble, data augmentation, and multitask learning techniques to improve overall performance and generalization.

Model ensemble methods enhance the overall prediction accuracy by integrating the predictions of multiple independent models, thereby reducing the bias and variance of individual models. For example, Qummar et al. [34] integrated five CNN models using stacking methods to extract salient features related to retinopathy. Additionally, certain studies have incorporated special preprocessing methods to enhance fundus images. For example, Nneji et al. [35] employed two independent deep learning models, InceptionV3 and VGG16, to process separate channels of input fundus images. The outputs of these models were weighted and merged to obtain the final retinopathy detection results. Kaushik et al. [33] proposed desaturation techniques in the preprocessing stage to address irregularities. They trained three CNN models concurrently and detected retinopathy by combining the optimal weights of these networks. These data augmentation-based preprocessing methods prevented the model from memorizing noise and training set details excessively, introducing stochasticity and diversity of transformations in the training data, and enabling the model to focus on key features and patterns of retinopathy in the fundus image.

On the other hand, Wang et al. [32] presented a method that simultaneously performed various tasks, including image resolution enhancement, lesion segmentation, and severity grading, to achieve high-precision retinopathy classification. Image resolution enhancement aided the model in capturing fine lesion details, lesion segmentation enabled the model to learn about the location and shape information of the lesion, while severity grading assisted the model in understanding different disease levels. Training in combination with these tasks allowed the model to acquire richer and more diverse knowledge. For each task, they employed a CNN-based approach with a powerful feedback mechanism utilizing the task-aware loss function. However, this approach increased model complexity and required more labeled data, resulting in increased training and inference time and resource consumption. Additionally, conflicts or interferences may arise between different tasks, making effective learning challenging.

To address these challenges, we propose a new method called CLRD. It fuses soft knowledge extracted from the CNN and Transformer models through a collaborative learning training paradigm to achieve efficient online distillation. Notably, under the CLRD method, all models maintain independent high-precision retinopathy detection capabilities. In terms of clinical impact, CLRD offers multiple advantages. It employs a collaborative learning strategy to integrate student models of different scales and architectures, extracts valuable pathology information from fundus images, and enhances model invariance by designing fundus image-specific aberration information to transfer knowledge and minimize generalization errors through knowledge transfer. To the best of our knowledge, no assisted learning-based online distillation method for retinopathy detection exists. Our CLRD method outperforms all other SOTA work in retinopathy detection performance, and hence can assist physicians in accurately diagnosing and treating retinopathy, thereby reducing patients’ pain and financial burden.

### 5.3. Limitation and Future Work

While our CLRD framework has shown promising results in detecting retinopathy using fundus images, there are still some limitations and opportunities for improvement. Firstly, although the CLRD framework can be applied to vision models of different scales and architectures, we only tested it on a limited number of models and did not comprehensively evaluate all possible models. Therefore, further exploration is needed to enhance model performance and generalization capabilities through knowledge-sharing and transfer among different models.

Secondly, while our CLRD framework has demonstrated promising results, more research is required to assess its real-world applicability and generalizability. It is essential to train and validate the framework on larger and more diverse datasets to enhance its performance and evaluate its effectiveness across different populations. Additionally, we suggest exploring alternative collaborative learning strategies and knowledge distillation approaches to further enhance the model’s performance and generalization abilities.

We believe that with further refinements and advancements in deep learning techniques, it is feasible to incorporate additional quantitative outputs into the CLRD framework. These outputs may include measures such as lesion severity grading, disease progression assessment, and individualized risk prediction. Such enhancements would allow for a more comprehensive assessment of retinopathy and provide valuable insights for clinical decision-making.

In summary, collaborative learning, as exemplified by the CLRD framework, represents the way forward in automated output for retinopathy detection. Its abilities to harness the collective knowledge of multiple models, enhance model invariance, and improve diagnostic performance make it a promising approach in the biomedical field. We believe that our study sheds light on the potential of collaborative learning and opens up avenues for further advancements in retinopathy detection.

## 6. Conclusions

In this study, we presented an online knowledge distillation framework for retinopathy detection, named CLRD. Our approach employed an ensemble strategy that enables vision models of various architectures and scales to learn valuable pathology information from fundus images through collaborative learning. Additionally, we included warped information specifically designed for fundus images to transfer knowledge and improve model invariance. For the retinopathy detection task, we selected two student models: the self-supervised pretrained Transformer-based BEiT and the CNN-based visual transfer model ConvNeXt. Through ablation experiments and comparative experiments on a fundus image dataset, we demonstrated that CLRD effectively reduced generalization errors while maintaining the independent predictive ability of the student models. Moreover, it achieved SOTA performance in retinopathy detection. Our findings suggested that the CLRD framework can significantly enhance the diagnostic ACC of fundus images, particularly in the detection of retinopathy.

## Figures and Tables

**Table 1:** ACC on fundus image validation set. The knowledge distillation framework is optimized by KL divergence loss.

Teacher Model	Teacher ACC	Student ACC
ResNet-50 [15]	86.30	76.33
ConvNeXt [13]	91.05	81.35
Vanilla ViT [16]	90.32	86.52
BEiT [12]	93.28	88.63

**Table 2:** Data distribution of fundus image dataset.

Source	Device	Annotator	Subject	Image	Normal	Retinopathy	Total
Ophthalmology	Canon, CR-2	(1, 2)	1014	2592 × 1728	912	452	1364
Consultation Center	NIDEK, AFC-330	(1, 2)	123	3000 × 3000	107	50	157
Overall	-	-	1137	-	1019	502	1521

Note: (1, 2) represents one retinal specialist and two ophthalmologists.

**Table 3:** Quantitative evaluation results of the CLRD student models; the results demonstrate the average metrics of the five-fold cross-validation.

CLRD Student	Type	ACC (%)	PRE (%)	REC (%)	SPE (%)	F1 (%)
BEiT [12]	Normal	98.77	98.31	99.88	96.51	99.09
	Retinopathy	98.77	99.74	96.51	99.88	98.10
	Average	98.77	98.78	98.77	97.62	98.76
ConvNeXt [13]	Normal	96.88	96.75	98.65	93.27	97.70
	Retinopathy	96.88	97.14	93.27	98.65	95.17
	Average	96.88	96.88	96.87	95.04	96.86

**Table 4:** Quantitative comparison results of different architectures as CLRD student models; the results demonstrate the average metrics of the five-fold cross-validation.

CLRD Type	CLRD Student	Params (M)	FLOPs (G)	ACC (%)	PRE (%)	REC (%)	SPE (%)	F1 (%)
CLRD-1	BEiT [12]	81.18	12.70	97.45	97.54	96.13	94.82	97.42
	ResNetV2 [15]	44.76	0.04	95.07	95.05	93.85	92.63	95.04
CLRD-2	Vanilla ViT [16]	102.44	16.88	97.12	97.19	95.83	94.53	97.09
	ConvNeXt [13]	50.18	8.68	95.64	95.86	93.52	91.39	95.56
CLRD-3	BEiT [12]	81.18	12.70	98.77	98.78	98.77	97.62	98.76
	ConvNeXt [13]	50.18	8.68	96.88	96.88	96.87	95.04	96.86

**Table 5:** Experimental results on sensitivity analysis of loss hyperparameter. The table summarizes the ACC (%) on the fundus image validation set under different hyperparameter settings. The results demonstrate the average metrics of the five-fold cross-validation.

*α*	0.1	0.2	0.4	0.6	0.8	1.0	1.5	2	2.5	5
BEiT [12]	96.13	97.95	98.00	98.27	98.62	98.77	98.92	98.85	97.63	97.20
ConvNeXt [13]	93.97	96.10	95.29	96.31	96.85	96.88	97.03	96.55	95.78	94.96

**Table 6:** Experimental results on sensitivity analysis of data augmentation policies. The table summarizes the ACC (%) on the fundus image validation set under different data augmentation policies. The results demonstrate the average metrics of the five-fold cross-validation.

Method	BEiT	ConvNeXt
Without Augmentation	95.13	94.29
Cutout	94.27	92.10
CLRD	98.77	96.88

**Table 7:** Quantitative comparison results with competitive SOTA models; the results demonstrate the average metrics of the five-fold cross-validation.

Model	Params (M)	FLOPs (G)	ACC (%)	PRE (%)	REC (%)	SPE (%)	F1 (%)
DenseNet121 [21]	7.90	2.83	90.14	90.11	90.12	85.13	90.03
ResNetV2 [15]	44.76	0.04	79.03	78.74	79.23	72.11	78.85
Xception71 [22]	42.33	9.88	91.47	91.91	91.42	84.28	91.21
EfficientNet [23]	19.22	1.50	92.45	92.89	92.41	85.77	92.24
Vanilla ViT [16]	102.44	16.88	77.77	77.70	77.71	71.97	77.70
MobileViT [24]	5.57	1.42	93.45	93.75	93.41	87.76	93.28
Swin Transformer [25]	109.07	15.19	67.26	67.05	67.19	58.27	67.13
ConViT [26]	86.39	16.81	79.32	79.04	79.31	72.28	79.15
CaiT [27]	46.82	8.63	78.67	78.23	78.67	65.95	77.61
EfficientFormer [28]	31.89	3.94	90.45	90.78	90.52	83.29	90.21
VOLO [29]	58.58	13.61	78.68	78.30	78.65	65.45	77.51
CLRD-BEiT [12]	81.18	12.70	98.77	98.78	98.77	97.62	98.76
CLRD-ConvNeXt [13]	50.18	8.68	96.88	96.88	96.87	95.04	96.86

**Table 8:** Quantitative comparison results with SOTA works for retinopathy detection on the EyePACS dataset. The table only summarizes the highest detection results reported in each work.

Work	Model	ACC (%)	PRE (%)	REC (%)	SPE (%)	F1 (%)
Xu et al. [30]	CNN	94.50	-	-	-	-
Pao et al. [31]	CNN	87.37	-	76.93	93.57	81.80
Wang et al. [32]	CNN	86.90	87.10	-	-	85.70
Kaushik et al. [33]	CNN	97.92	-	97.77	100.00	-
Qummar et al. [34]	CNN	80.80	63.80	-	86.70	53.70
Nneji et al. [35]	InceptionV3 and VGG16	98.00	-	98.70	97.80	-
Our Proposed	CLRD-BEiT	99.05	98.83	99.12	98.79	98.28
Our Proposed	CLRD-ConvNeXt	98.60	97.13	98.25	98.86	97.32

## Data Availability

The data presented in this study are available on request from the corresponding author. The data are not publicly available due to the privacy of the patients.
